# Unilateral Cataract and Vitreoretinopathy in a Case with Klippel-Trenaunay Syndrome

**DOI:** 10.1155/2014/312030

**Published:** 2014-06-16

**Authors:** Osman Okan Olcaysu, Ahmet Altun, Elif Olcaysu, Ebru Marzıoğlu Ozdemır, Berrin Demır

**Affiliations:** ^1^Clinic of Ophthalmology, Erzurum Training and Research Hospital, 25240 Erzurum, Turkey; ^2^Clinic of Ophthalmology, Fatih Sultan Mehmet Training and Research Hospital, 34752 Istanbul, Turkey; ^3^Department of Pediatrics, Medical Faculty, Ataturk University, 25240 Erzurum, Turkey; ^4^Department of Medical Genetics, Erzurum Training and Research Hospital, 25240 Erzurum, Turkey; ^5^Department of Radiology, Erzurum Training and Research Hospital, 25240 Erzurum, Turkey

## Abstract

*Purpose*. We present a case with Klippel-Trenaunay (KT) syndrome that had unilateral mature cataract and vitreoretinopathy. *Case Report.* A 17-year-old boy with KT syndrome presented to the clinic of ophthalmology for low vision in the right eye. His best corrected visual acuity (BCVA) was hand motion in the right eye and 20/20 in the left eye. Anterior segment examination revealed mature cataract in the right. During the physical examination, port-wine stains were noted over right side of his face, ankle, and toes. He had asymmetric face and his head was larger on the right side. Leg lengths were symmetrical, although he had skin hypertrophy. Cranial magnetic resonance imaging studies showed cortical atrophy discordant to his age, asymmetric vascular dilatations in the right hemisphere, hypertrophy in the right periorbital soft tissue, and choroidal plexus. The patient received an uncomplicated cataract surgery. His BCVA in the right eye improved to 20/200 after the surgery. After removing cataractous lens, we were able to examine the fundus that revealed severe vitreoretinopathy and choroidal hemangioma. *Conclusion.* This case emphasizes the importance of prompt ophthalmic examination in patients with KT syndrome.

## 1. Introduction

Mostly known regional overgrowth syndromes are Klippel-Trenaunay (KT) syndrome, Proteus, Maffucci syndromes, and neurofibromatosis. Klippel and Trenaunay first described KT syndrome in 1900 [1]. In 1918, Weber included arteriovenous fistulae in this overgrowth disorder, and these pathological conditions have been grouped ever since under the name of Klippel-Trenaunay-Weber (KTW) syndrome [2]. Regional hypertrophy in KT syndrome occurs usually in extremities, internal organs, peripheral nerves, and the brain. Pathogenesis of the KT syndrome is still remaining unclear [1, 2].

Here in, we would like to present a case with KT syndrome that had unilateral mature cataract and vitreoretinopathy. This report is unique, because it is the first case of KT syndrome accompanied with mature cataract and vitreoretinopathy in the literature.

## 2. Case Report

**Figure 1:**
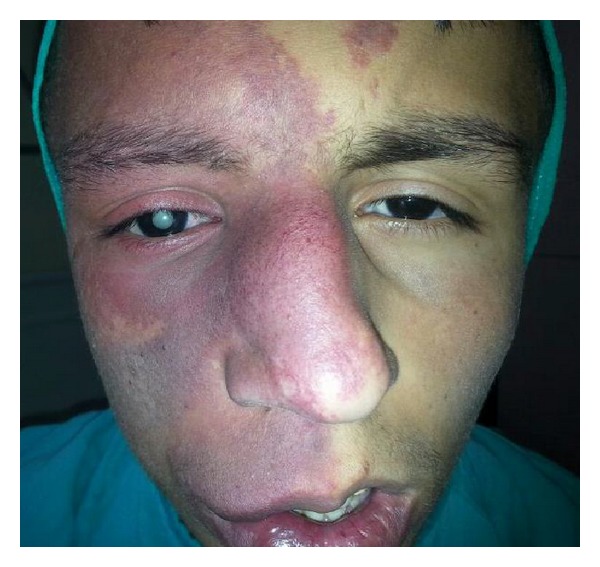
Mature cataract, port-wine stains, and asymmetric face in the right side.

A 17-year-old boy with the features of KT syndrome presented to the Clinic of Ophthalmology of Erzurum Region Education and Research Hospital with the complaint of low vision in the right eye. His mental status was adequate to communicate without trouble. Ocular alignment was orthophoric, and extraocular muscle movements were not limited. Corneal topography revealed normal pattern bilaterally. His best corrected visual acuity (BCVA) was hand motion in the right eye and 20/20 in the left eye. Anterior segment examination revealed mature cataract in the right eye (Figure 1) and normal findings in the left eye. Funduscopic examination was within normal limits in the left eye. There was no determined retinal detachment but increased choroidal thickness in ocular ultrasonography scan of the right eye.

**Figure 2:**
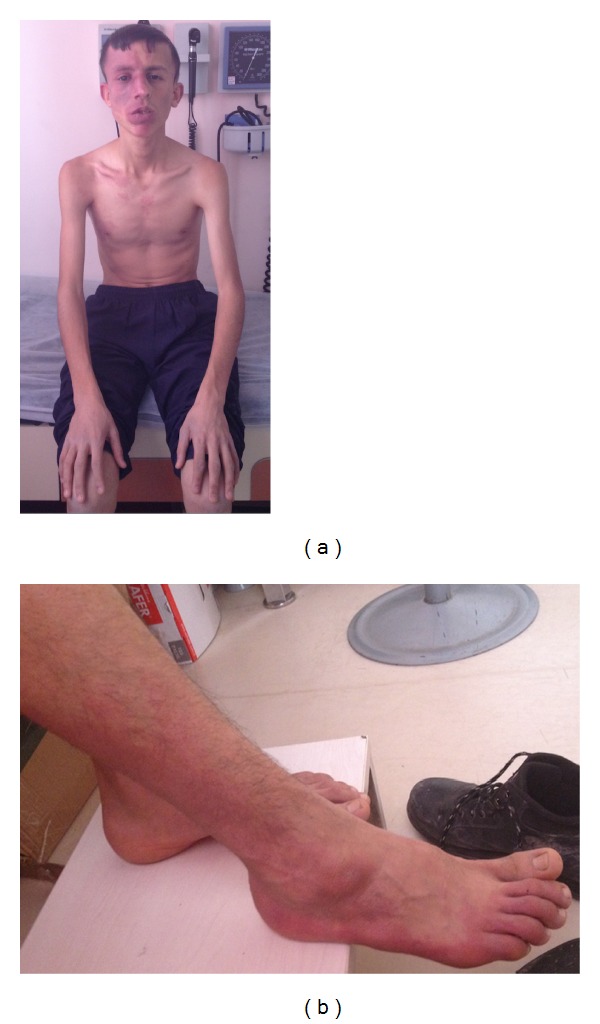
Port-wine stains over the right side of his face, ankle (a), and toes (b).

According to the history obtained from his parents, he was born following uncomplicated vaginal delivery. The parents reported a congenital cutaneous vascular malformation on his face with the color of purple at birth. Family history was negative for KT syndrome and other phakomatoses. During the physical examination, port-wine stains were noted over the right side of his face (Figure 2(a)), ankle, and toes (Figure 2(b)). His face was asymmetric and his head was larger on the right side. Leg lengths were symmetrical, although he had skin hypertrophy. The hair and teeth were appearing normal. The remaining of general and neurological examinations was within normal limits. Psychomotor development was apparently normal. Electrocardiogram, leg X-rays, and heart, abdominal, and pelvic ultrasound examinations were normal.

**Figure 3:**
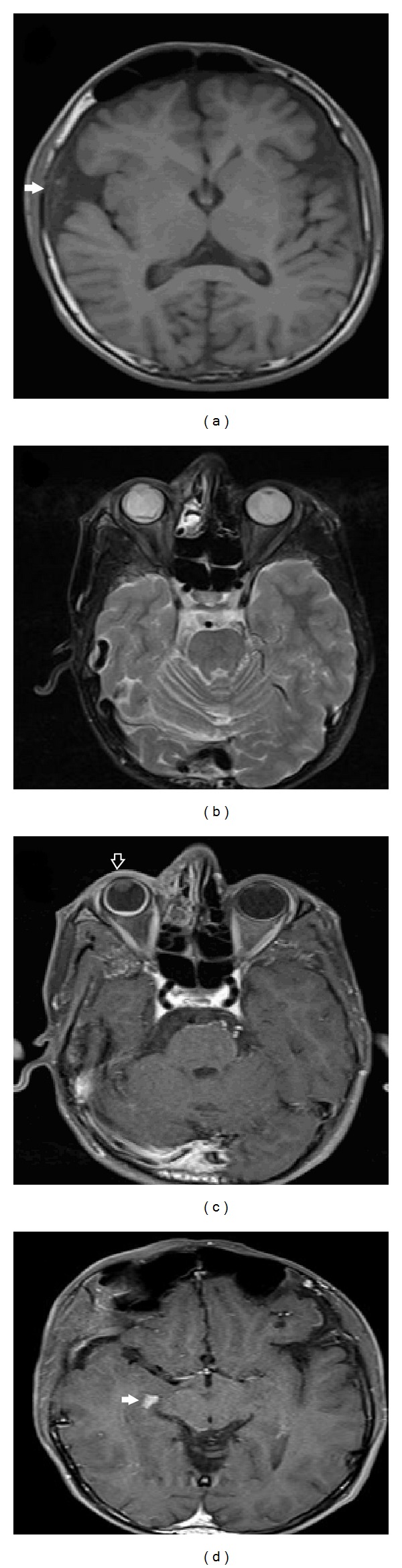
Cranial magnetic resonance imaging studies showed cortical atrophy discordant to his age (a), asymmetric vascular dilatations in the right hemisphere (b), hypertrophy in the right periorbital soft tissue (c), and choroidal plexus (d).

Cranial magnetic resonance imaging (MRI) studies showed cortical atrophy discordant to his age (Figure 3(a)), asymmetric vascular dilatations in the right hemisphere (Figure 3(b)), hypertrophy in the right periorbital soft tissue (Figure 3(c)), and choroidal plexus (Figure 3(d)).

**Figure 4:**
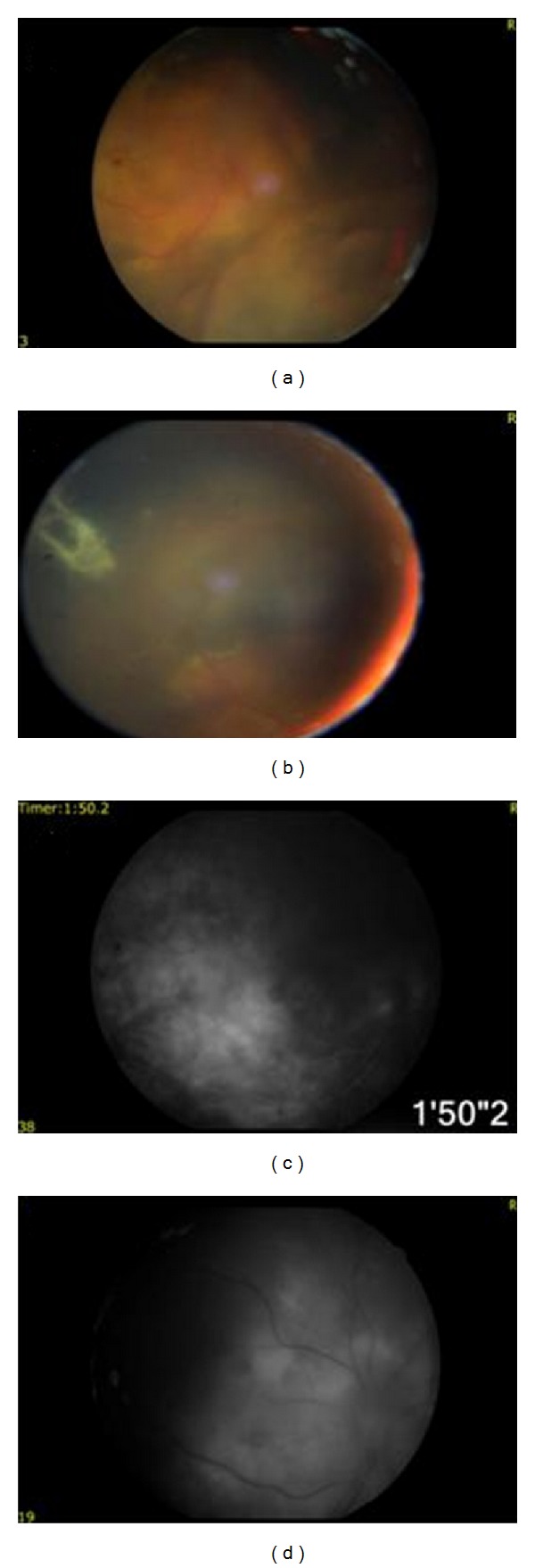
After cataract surgery, funduscopic examination revealed severe vitreoretinopathy (a) and choroidal hemangioma (b) in the right eye. Fundus fluorescein angiography demonstrated leaking from dilated retinal vessels (c), ischemia, and neovascularization (d).

**Figure 5:**
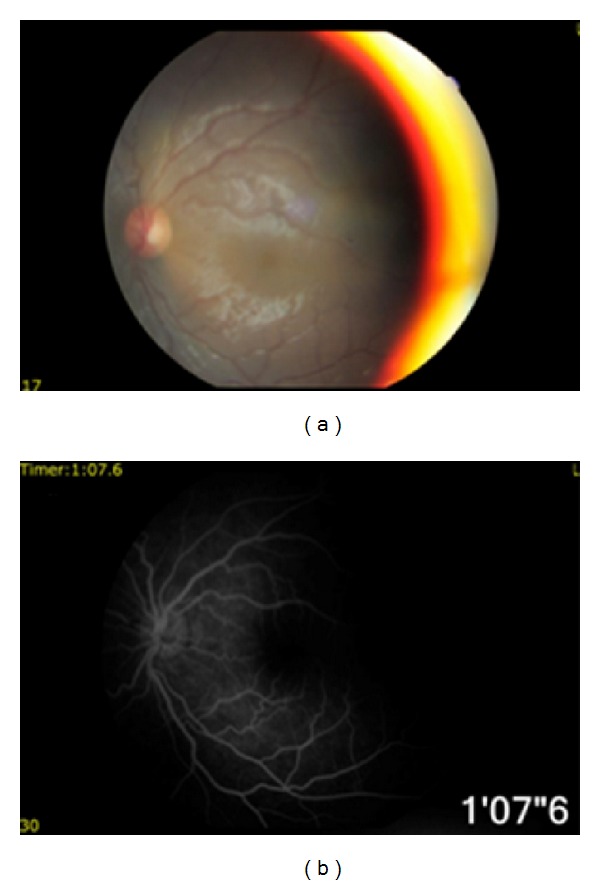
Funduscopic examination (a) and fundus fluorescein angiography (b) findings were within normal limits in the left eye.

The patient received an uncomplicated cataract surgery under sub-Tenon's local anesthesia by phacoemulsification for the right eye. His BCVA in the right eye improved to 20/200 after the surgery. After removing cataractous lens, we were able to examine the fundus that revealed severe vitreoretinopathy (Figure 4(a)) and choroidal hemangioma (Figure 4(b)). Fundus fluorescein angiography findings revealed dilated retinal vessels (Figure 4(d)), neovascularization, leaking (Figure 4(c)), and ischemia in the right eye. The funduscopic examination (Figure 5(a)) and fundus fluorescein angiography (Figure 5(b)) findings were within normal limits in the left eye.

## 3. Discussion

KT syndrome is one of the least common syndromes of the phakomatoses affecting ocular structures. Ocular and neurological abnormalities in KT syndrome are more common when the cutaneous angiomas involve the face. Limb anomalies, positional limb and/or trunk defects, autonomic dysfunction, and various systemic complications have been also reported with KT syndrome [3].

Ophthalmic features of KT syndrome are orbital varix, retinal varicosities, choroidal angioma, melanoma, persistent fetal vasculature, heterochromia iridum, and glaucoma [4]. Several fundus changes have been reported in the cases of KT and KTW syndromes [5]. The most frequently reported fundus finding is diffuse choroidal hemangioma that is often ipsilateral to a facial cutaneous hemangioma [5], as was in our case.

Good and Hoyt [6] reported a case of a 10-year-old girl with KTW syndrome that had enlargement of optic nerve shadow. Spoor et al. [7] reported an 18-year-old girl with KTW syndrome that had bilateral optic nerve meningiomas [7]. Optic nerve hypoplasia and tilted optic nerves have also been also documented [8] in the literature.

Our patient was displaying typical systemic findings of KT syndrome, including vascular nevus involving extremity, peripheral varicosities, and hemihypertrophy of soft tissue. This case did not have KTW syndrome because of the absence of arteriovenous malformation. This report is unique, because to our knowledge it is the first reported case of KT syndrome accompanied with unilateral mature cataract and vitreoretinopathy in the literature.

## 4. Conclusion

This case emphasizes the importance of prompt ophthalmic examination in patients with KT syndrome.
